# Poisoning by Strychnine

**Published:** 1866-12

**Authors:** S. A. McWilliams

**Affiliations:** Chicago


ARTICLE XLVI.

### POISONING BY STRYCHNINE.

By S. A. McWILLIAMS, A.M., M.D., Chicago.

On March 15th, 1866, Mr. T., married, of sanguine temperament, *æt.* 31, took 5 grs. strychnine, at 15 minutes before 1 o'clock A.M., with the intention of poisoning himself, on account of financial and family troubles, real or imaginary. Immediately after taking the poison, he felt a burning sensation in his stomach for about a minute, and then felt as if his blood grew cold. At 1 o'clock, he went to bed and soon fell asleep; at 2 o'clock, he vomited; at 2½ o'clock, he tried to get out of bed, but found himself too stiff to do so. Mr. S., with whom he was sleeping, gradually became conscious of an unusual tremulous motion of the spring-bed, and of the random spitting of his bed-fellow. Learning the cause of the trouble, upon inquiry, Mr. S. ran for a physician, and stated the cause of alarm. The writer reached the patient at 3½ o'clock, and found him having spasms, extensive, frequent, and severe, and, with each, a blowing of froth from his mouth. He lay upon his back; arms extending obliquely from his body; face flushed; perspiration rolling off him; pupils dilated widely; pulse 130 per minute; color of lips natural; no lividity of any part; complaining of stiffness of muscles and inability to move limbs; no pain in head; no vertigo; no tinnitus aurium; mind exceedingly clear and perfectly sensible. Some of the matter vomited and the articles used in preparing the potion were secured. A drachm of the tr. of cannabis indica was immediately given, and another in 5 minutes; then two similar doses at intervals of 10 minutes; afterwards, two such doses, at intervals of 15 minutes, with a rapid amelioration of the symptoms; the next drachm was given at 5 o'clock. The patient was now hopeful, talkative, and cheerful. Slight spasms occurred at intervals of 5 minutes, with tremulous movements running through the system between. In consultation, it was now decided best to give only 15 drops of tr. of cannabis indica, at intervals of an hour.

At 6½ o'clock, I went home, leaving my patient apparently out of danger, but was again hastily summoned, at 8½ o'clock, to find him, to every appearance, in a worse condition than at first. Soon after I left, he had vomited copiously. Two drachms of his previous medicine were immediately given him, and in 10 minutes after, a drachm of tr. of camphor.

I requested Dr. N. S. Davis to see the patient with me. He did so at 9 o'clock, and advised that drachm doses of the tr. of cannabis indica and tr. of camphor be alternated at intervals of 15 minutes, and, as his symptoms improved, to gradually lengthen the intervals.

From 8½ to 12 o'clock, the patient was slightly delirious; had spasmodic contraction of the muscles of the chest about four times in a minute, with a feeling of impending suffocation. At 10 o'clock, pupils less dilated; pulse 100. At 11 o'clock, pulse 90; a violent spasm occurred every 15 minutes, and about four thrills per minute running through the system. At 11½ o'clock, the patient vomited. At 12 o'clock, pulse 100; pupil as before; tongue coated; spasms rare and slight. Took a mouthful of water for the first. Lengthened interval between medicine to half-an-hour. Up to this time, there was increased sensitiveness to external impressions, so that the spasms, like electric shocks, would ensue upon the slightest causes, as the touching of the bed; contact of a spoon upon the lips; a sudden step; a jarring of the room, or slightest noise. About 1 o'clock P.M., he had two or three slight spasms at longer intervals. At 3½ o'clock, pulse 120; pupil natural, and continued so; lips dry; perspiration has ceased, but skin still moist; inclined to sleep; complains of soreness of muscles. At 5 o'clock, pulse 100; medicine as before. At 9 o'clock, pulse 100; lips dry; ordered toast-water; spasmodic contraction of chest gone; able to move arms comfortably; passed urine for the first. Half-drachm doses of medicine at intervals of half-an-hour, and lengthen gradually to every hour. On the 16th, at 7 o'clock pulse 95; the lips were still dry; he had rested comfortably and slept some during the night; a half-drachm dose of the medicine ordered every 90 minutes; has lain in first position, on back, without any desire to turn on either side; ordered tablespoonful of gruel every half-hour. 11 o'clock, pulse 90; patient much brighter than in the morning; lips and skin natural; omitted camphor, and continued half-drachm doses of cannabis indica every two hours; directed a tablespoonful of soup and one of gruel every half-hour. 7 o'clock, pulse 80; tongue still coated; directed to give medicine at 9 P.M., 1 A.M., and 6 A.M., as bowels had not moved. On the 17th, at 7 A.M., pulse 70; slept well during the night; passed urine during the night and in the morning; appetite returning; medicine discontinued; toast and tea given; directed citrate of magnesia, until bowels should move, in doses of three fluid ounces, every three hours. 7 P.M., pulse 65; skin natural; bowels moved; appetite fair; sat up to have bed made. 18th, 2 P.M., patient up and dressed; slept well during the night; feels perfectly well, only very weak. Convalescence was uninterrupted.

Prof. F. Mahla kindly applied such tests to the suspected substances as left no doubt as to the poison being strychnine. The residue left in the tumbler was of an intensely bitter taste, and upon application of a small amount of concentrated sulphuric acid there was no change of color, but on the addition of a small piece of bichromate potassa a succession of colors ensued, gradually fading out—purple, blue, deep red, reddish brown, yellow, etc.

The muscles of the lower jaw in the above case were not affected, while in idiopathic or traumatic tetanus they are usually affected first. The violence of the shock to the nervous centres, in this case, seem to have blunted, to an extraordinary degree, their susceptibility to narcotic influence.

